# An economic analysis of a system wide Lean approach: cost estimations for the implementation of Lean in the Saskatchewan healthcare system for 2012–2014

**DOI:** 10.1186/s12913-017-2477-8

**Published:** 2017-08-03

**Authors:** Nazmi Sari, Thomas Rotter, Donna Goodridge, Liz Harrison, Leigh Kinsman

**Affiliations:** 1 Department of Economics, University of Saskatchewan, Arts 815, 9 Campus Drive, Saskatoon, SK S7N 5A5 Canada; 2 College of Pharmacy & Nutrition, University of Saskatchewan, Saskatoon, SK S7N 5A5 Canada; 3 College of Medicine, University of Saskatchewan, Saskatoon, Canada; 4 School of Physical Therapy, College of Medicine, University of Saskatchewan, Saskatoon, Canada; 5 University of Tasmania and Tasmanian Health Organisation (North), Launceston, TAS Australia

**Keywords:** Lean, Lean activities, Implementation cost, Cost estimates, Economic evaluation, Quality improvement

## Abstract

**Background:**

The costs of investing in health care reform initiatives to improve quality and safety have been underreported and are often underestimated. This paper reports direct and indirect cost estimates for the initial phase of the province-wide implementation of Lean activities in Saskatchewan, Canada.

**Methods:**

In order to obtain detailed information about each type of Lean event, as well as the total number of corresponding Lean events, we used the Provincial Kaizen Promotion Office (PKPO) Kaizen database. While the indirect cost of Lean implementation has been estimated using the corresponding wage rate for the event participants, the direct cost has been estimated using the fees paid to the consultant and other relevant expenses.

**Results:**

The total cost for implementation of Lean over two years (2012–2014), including consultants and new hires, ranged from $44 million CAD to $49.6 million CAD, depending upon the assumptions used. Consultant costs accounted for close to 50% of the total. The estimated cost of Lean events alone ranged from $16 million CAD to $19.5 million CAD, with Rapid Process Improvement Workshops requiring the highest input of resources.

**Conclusions:**

Recognizing the substantial financial and human investments required to undertake reforms designed to improve quality and contain cost, policy makers must carefully consider whether and how these efforts result in the desired transformations. Evaluation of the outcomes of these investments must be part of the accountability framework, even prior to implementation.

**Electronic supplementary material:**

The online version of this article (doi:10.1186/s12913-017-2477-8) contains supplementary material, which is available to authorized users.

## Background

Improving safety and quality of healthcare systems while controlling costs is a key aim of healthcare systems around the world [1–4]. Process management theory suggests that the best way to reduce costs is to improve quality [5]. The costs of undertaking health care reforms to improve quality, however, are often underestimated, with most of the cost savings realized by improving “upstream” care to avoid “downstream” failures and recovery costs [6, 7]. Industrial solutions such as Lean are now being widely implemented in health care to reduce waste, enhance value, and improve quality [8].

Lean is a management system originating over 50 years ago as the Toyota Production System, a method to improve production by eliminating waste [8]. In the field of operations management, the most frequently cited definition of Lean is offered by Shah and Ward, who define Lean as a complex intervention in the form of ‘an integrated socio-technical system to eliminate waste by concurrently reducing or minimizing supplier, customer, and internal variability’ [9]. In healthcare, Lean management is often described as a continued commitment to its principles and continuous quality improvement based on the application of Lean activities to identify waste and sustain changes [8, 10]. Waste in healthcare is defined as activities not adding value for the patient, such as wait times.

The Saskatchewan Ministry of Health (MOH) has made a multi-million-dollar investment in a massive overhaul of the provincial healthcare system to produce ‘better health, better value, better care, and better teams.’ The initial phase started with a project using Releasing Time to Care (RTC) in acute care medical and surgical wards in Saskatchewan, Canada in 2010 [11]. In 2012, the province decided to use a comprehensive Lean approach, engaging a consulting firm to assist with the Lean implementation across the entire healthcare system in the province [12]. The initial phase of Lean implementation focused on leadership training, and the creation of Kaizen Promotion Offices (KPOs) that provide supportive infrastructure to coordinate and advance the Lean transformation. While some of the costs involved in this investment, such as payment for consulting services, are known, other costs involved in training hundreds of health system leaders and implementing Lean events across the province have not been well documented.

Although Lean has been widely applied within Canada and internationally [10, 11, 13–16], published studies have not focused on cost estimates for activities and processes used to implement Lean management in health care [15, 17, 18]. The aim of this study was to address this gap by estimating the costs of the initial phase of Lean implementation in the province of Saskatchewan (2012–2014 period). While doing this, direct and indirect costs were estimated for each type of selected Lean implementation activity.

A number of Lean activities has been implemented across the health care sector in other parts of the world. However, compared to the Saskatchewan experience, none of those reported has implemented a comprehensive set of Lean philosophies, processes and activities. The most frequently reported processes and activities in the literature to implement Lean in healthcare are 5S events to reorganize the workplace, rapid process improvement workshops (RPIW), and value stream mapping (VSM) to improve current and future care processes [18–22]. In Saskatchewan’s Lean implementation, each of these activities and processes has been used extensively [8]. Five main Lean processes and management activities (RPIWs, 5S, Kaizen basics workshops, mistake proofing projects, and Kanban events) have been widely implemented across the province [8]. It is therefore essential to estimate the costs associated with the implementation of various kinds of activities and processes in order to evaluate the return from Lean in Saskatchewan. We describe each activity below with specific reference to its implementation in Saskatchewan.

## Lean activities in Saskatchewan

As part of the transformation of the healthcare system in Saskatchewan, five main Lean processes and management activities were widely implemented. These are briefly described below.

### Rapid process improvement workshops

RPIWs are multi-week, highly detailed interventions in which value stream mapping and 5S take place three weeks and five weeks prior to the final week of the event. The main purposes of an RPIW are to increase efficiency and to improve patient safety and care [8]. These workshops are time intensive activities, which require participation by the team lead, the sub-team lead, KPO support personnel and several other team members. The team members are recruited to the activity from the unit in which the RPIW is implemented. Although a typical RPIW is expected to include five to six team members (including patient advisors, who are compensated for their time), it is likely that there was considerable variability in implementation across the province.

### 5S event

5S stands for sorting, simplifying, sweeping, standardizing and self-discipline [8]. It is an activity for arranging organizing the workplace to make it clean, neat and well organized with the purpose to eliminate waste, promote teamwork and safety, decrease the cost, and improve the productivity. As it is implemented in Saskatchewan, a 5S lasts one week (37.5 work hours), during which one team lead, the sub-team lead, KPO support personnel, and team members participate in the event.

### Kaizen basics workshops

Kaizen basics workshops are training workshops with the purpose of introducing the basic concepts of Lean as a continuous quality improvement approach and exposing the healthcare staff to Lean terminology. It takes one workday with KPO support personnel (a Kaizen specialist) participating as an instructor/facilitator of the event.

### Mistake proofing projects and the North American Tour

Mistake proofing projects are focused projects with the purpose of eliminating defects and errors in the healthcare system [8]. Examples include a project to prevent contamination of specimens in a laboratory test and another aimed at increasing patient safety by eliminating the errors in labelling of tests or samples.

A North American Tour (NAT) is a 5-day training tour to learn how to organize and lead mistake proofing projects through observation and meetings with other organizations that are well-known examples of successful implementation of Lean. During a NAT tour, participants spend one day at AutoLiv Lean Manufacturing (Ogden, Utah), two days at the Virginia Mason Institute (Seattle, Washington), and half a day each at Seattle Children’s Surgery and Ambulatory Clinic (Seattle, Washington). The tour participants leave the province on Sunday for Utah, and return on Friday from Seattle. The tour including travel time takes five full days with an average of 20 participants in each. The participants in mistake proofing and the NAT are the Lean leader trainees. They are either middle to upper level managers (CEOs, vice-presidents, or directors) or Kaizen support personnel. Therefore, in the costing described in the next section, we assume that their average wage is the same as government managers.

### Kanban management

Kanban is a management activity for effectively implementing the just-in-time (JIT) production system to make sure that required supplies and materials become available when and where they are needed [8]. The goal of this activity is to improve the flow of work or materials through the process system while pursuing the JIT delivery model, reduce the excess inventory, and develop a reliable inventory management process. To be effective, there must be timely review of the inventory levels and the routine estimation of the reorder to have smooth flow of supplies and materials.

## Method

In this section, we describe the data and the methodology related to the estimation of cost for the Lean implementation in Saskatchewan. As part of the cost, we compute the direct and indirect cost of Lean implementation activities. These data sources and methods are described in the subsections below.

### Data and data sources

In order to obtain detailed information about each type of Lean events, as well as the total number of corresponding Lean events, we used the Provincial Kaizen Promotion Office (PKPO) Kaizen database [23]. Although the database has been updated on a regular basis, it is possible that there were more events than the ones included in the database. As a result, the estimated total cost of Lean for two years may be underestimated. As explicitly noted in section 3.2.2, we made plausible assumptions when we did not have the specific information that was needed. In these instances, we chose to make conservative assumptions not to overestimate the cost of Lean. The readers should consider this when making inferences based on our estimates.

In order to quantify the number of hours devoted to each Lean event, we reviewed the corresponding project forms available at the PKPO Kaizen database [23]. For RPIWs, a typical event is expected to include five to six team members, but there may be considerable variability in implementation across the province. To understand the ways that RPIWs they were implemented in Saskatchewan and to estimate the average number of participants in RPIWs, as well as their job titles, we reviewed the RPIW project forms for all RPIWs that took place in the four largest health regions in the province (Saskatoon Health Region [SHR], Regina Qu’Appelle Health Region [RQHR], Five Hills Health Region [FHHR], and Prince Albert Parkland Health Region [PAPHR]) during 2013. Based on our review of 87 RPIWs, we documented the roles and corresponding total hours of each participant during the course of a typical RPIW event. The details presented in Table 1 show that an RPIW requires a total of 633 work hours (equivalent to 84.4 work days).

**Table 1:** Time commitment and roles of participants in a typical Lean activity (hours per event)

	RPIW	5S	Kaizen basics workshops	Mistake proofing	Kanban
Team leader	112.5	37.5	-	120	90
Sub-team leader	103.1	37.5	-	120	90
KPO support	159.4	37.5	7.5	-	75
Lean leader participant	45.7	1.0	-	-	-
Physician	15.5	3.1	3.2	-	-
Patient representative	40.1	-	-	-	-
Other participants	156.5	99.0	225	562	279
Total					
Hours	632.8	215.6	235.7	802	534
Work days	84	29	31	107	71

Source: Our own computations using the project forms for the corresponding Lean events [23]. These estimates were computed based on the implementation of the activities in the 2012–2014 period

For 5S events, we reviewed the project forms for thirty-six events held in four health regions (SHR, RQHR, FHHR, PAPHR) during 2013. There were variations in the number of participants in each event. Although most events require five to six team members, there were a few events with two to three team members. Due to this variation in number of team members, we computed the average number of team members based on thirty-six 5S events and documented hours of participation, along with the designations of team members. As presented in Table 1, a typical 5S event requires 216 work hours, which is equivalent to about 29 workdays.

The number of participants in each Kaizen basics workshop ranged from eight to 75 with an average number of 30 individuals. The goal of the Saskatchewan Ministry of Health was to train about 40,000 healthcare workers through one-day workshops during the first phase of the Lean implementation. From April 2012 to March 2014, there were 16,963 healthcare workers, including 244 physicians, who participated in these workshops. A typical workshop was attended by 30 healthcare staff and a facilitator. As a result, a Kaizen basics workshop takes about 236 h, equivalent to 31 work days (Table 1).

From start to end, a mistake proofing project lasted about 28 weeks with occasional participation by the project participants during the course of the event. Team and sub-team leads and team members participated in the projects. Mistake proofing projects occurred locally or in some cases as part of the Lean Leader Certification training. When a mistake proofing project was done as part of Lean Leader Certification training, all project participants attended a NAT during the 13th week of the mistake proofing project. In this situation, the team and sub-team leads spent 75 h before and 45 h after the NAT on the project, whereas the team members spent 72 h before and 45 h after the NAT. While the number of team members varied from one project to another, the average number of team members for an event was 4.8 individuals. Table 1 presents a summary of the hours required to carry out a local mistake proofing project. In addition to time required for the NAT, a typical mistake proofing project requires 802 h of work, which is equivalent to 107 workdays.

As presented in the PKPO Kaizen database, a typical Kanban activity involves several participants, including the team and sub-team leads, as well as KPO support personnel and a coordinator. Similar to the other Lean activities, a Kanban includes several team members ranging from two to eight. Most Kanban activities that took place until April 2014 had more than five team members. We used all 33 Kanban events that took place in Saskatchewan until April 2014 and computed the average number of team members for a Kanban activity [23]. This average was 6.03 individuals with each member expected to participate for 37.5 h during the course of the event. As presented in Table 1, a Kanban, therefore, requires a total of 534 h of work, equivalent to 71 work days.

### Data analysis

#### Time cost of Lean activities

The indirect cost of Lean implementation is primarily the cost of personnel time during participation in Lean events. As a general rule, we estimated the opportunity cost of participants’ time devoted to the events using their market wage rate [24]. The exact wage rate for each participant was not available; therefore, we used an approximate wage based on their job titles and affiliations [24]. Specifically, we used low (<25th percentile), average, and high (>75th percentile) wage rates reported in the 2011 Saskatchewan Wage Survey [25] after adjusting them for the Saskatchewan inflation rate of 1.6% in 2011–2012 and further by 1.5% in 2012–2013 [26].

When we obtained the relevant wage rates for the corresponding Lean event participants, we used the job titles and affiliations reported in the project forms for the relevant Lean events. For participants with unknown designations and job titles,^1^ we used the Saskatchewan average wage rate in the healthcare sector. Furthermore, for those Lean event participants with the roles of team leader, sub-team leader, Lean leader participants, or Kaizen support, we used the government manager wage rates in the healthcare sector.^2^

Given that the Saskatchewan Wage Survey does not include employer paid benefits or other contributions, we included these contributions and benefits (Canadian Pension Plan [CPP] and employment insurance [EI] contributions; workers’ compensation premiums; supplementary pension plan [e.g., RRSP] contributions; health, vision and dental care premiums) with the gross wages. The EI and CPP contributions paid by the employer are 2.632% and 4.95% of income up to a maximum income of $47,700 and $51,100^3^ respectively [27, 28]. We used a workers’ compensation premium of 2.04%, as reported by the Saskatchewan Workers’ Compensation Board for the industry group G22 [29].^4^ We used 6% as the supplementary pension plan contribution paid by the employer and a total of $2500 per year for other health benefits (such as dental and vision care and prescription drugs).^5^

In order to account for additional costs due to overtime payments, we assumed that backfilling was only carried out for clinical personnel (e.g., nurses). Our assumption was based on our research team’s qualitative interviews suggesting that only front-line nurses were backfilled because of their responsibilities for direct and critical patient care [30]. Based on our review of RPIW project forms, we determined that the participation by nurses in a typical RPIW was a total of 35 h. Therefore, the cost of backfilling for nurses was $800 for each RPIW. For other events, we did not have detailed information in terms of numbers of nurse participants; therefore, we computed corresponding overtime compensation costs for other events using the relative time of the corresponding event compared to an RPIW as weights (i.e., proportion based on time).

### Direct cost of Lean activities

The direct cost of Lean is the second component that needs to be taken into account. The major contributing factor to the direct cost is consulting fees paid to John Black and Associates (JBA) during the course of the Lean implementation.

Another direct cost of Lean is the physician remuneration, as well as the honoraria paid to the patient/family representatives. Given that most of the physicians in the province are paid by fee-for-service, they have been compensated for their time devoted to the Lean events. Although there is a fixed remuneration of $187.50 per hour for physicians, honoraria for patient/family representatives vary by health region from a minimum of $70 to a maximum of $200 a day [31]. Therefore, we used three levels of patient/family representatives’ honoraria: low ($70/day), average ($135/day), and high ($200/day).

Training of Lean leaders is integral to the implementation of Lean. In the qualitative sub-study [30], participants clearly articulated the pivotal importance of ensuring that leaders responsible for Lean implementation were well-versed in Lean philosophy and activities. As noted previously, some events to prepare Lean leaders, such as the NAT, involve significant travel, accommodation, and other incidental expenses. These and other related expenses (e.g., expenses for printing and supplies) are additional direct costs that are included in our estimations. When relevant for a specific type of Lean event (e.g., RPIWs, 5S, or Kaizen basics workshops), we assumed a $100 weekly average cost for printing and supplies.^6^

Following the provincial travel guidelines, we used a weekly cost of $1500 per person for travel, accommodation, and other incidental expenditure within the province [32]. In the case of Kaizen basic workshops, it is likely that the small health regions did not have a trained Kaizen lead specialist to facilitate the workshops. Therefore, these regions may need this service delivered by PKPO personnel. As a result, there might be additional travel and accommodation costs for each workshop in these regions.

Our review of the workshop database showed that half of the participants trained in the workshops are from the SHR, RQHR or PAPHR. As a result, only half of the training workshops (i.e., all workshops in small health regions) required one-day of travel by the Kaizen lead specialist from the PKPO. In other words, there was a one-day travel and accommodation expense for two workshops; this suggests that the travel related costs for the Kaizen lead specialist was 50% of the daily travel cost for each session, leading to a $150 travel related cost per session.

The details for the average cost of a NAT with an average of 20 participants are presented in Table 2. We used the provincial travel guidelines for government business [32]. The airfare was computed based on economy class tickets using online databases (e.g., expedia.ca). Our search showed that the round trip airfare from Saskatoon was $621.^7^ Additional expenses were computed for local transportation using Saskatoon taxi rates. This means a $25 local transportation cost per person for three round trips to the airports. Accommodation expenses were estimated based on our review of online databases (.e.g., expedia.ca) for hotel rates in the corresponding cities in the US. The total cost of a tour reported in Table 2 also included the accommodation cost based on five-night stays at an average of $140/night per person. For the cost of food, we used the standard per-diem rate of $51 per person for 6 days [33].

**Table 2:** Average cost for selected Lean activities (in CAD$)

	RPIW	5S	Kaizen basics workshop	Mistake proofing	NAT	Kanban
Participants’ time cost	29,361	10,206	8304	45,676	42,735	22,999
Physician remuneration	2909	587	484	n/a	n/a	n/a
Patient/Family representative honorariums	721	n/a	n/a	n/a	n/a	n/a
Printing/supplies	200	100	100	n/a	n/a	n/a
Travel/accommodation	1500	n/a	150	n/a	27,920	n/a
Per-diems	n/a	n/a	n/a	n/a	6120	n/a
Overtime compensations	800	272	299	1016	152	676
Total	$35,491	$11,165	$9337	$46,692	$76,927	$23,675

Source: Our own computations

n/a stands for not applicable

Another source of direct costs was the operational cost of the KPOs. After the beginning of Lean implementation in Saskatchewan, Quality Improvement Departments (QIDs) in health regions were transformed into KPOs with Quality Improvement (QI) personnel transferred to the new offices. This transformation by itself may not have generated additional costs that could be attributable to the Lean initiatives if the KPO size and other KPO related expenses stayed the same compared to pre-Lean period. However, there were health regions without previous QIDs where KPOs were launched following the Lean initiative in the province. It is also possible that the number of employees of the KPOs may have become larger than that of the QIDs. Therefore, the differences in number of employees and other expenses between previous QIDs and current KPOs need to be compared in order to account for additional costs related to the change in QI teams.

It is our understanding that the QI teams were relatively more fragmented during the pre-Lean period. Some health regions with no formal QIDs had implemented QI work with their existing personnel. These employees who were transferred to the new KPOs cannot be regarded as additional KPO related costs. If we do not account for the employees who were not located in the QIDs but doing QI work, we would overestimate the cost of Lean. Hence, we should estimate the full time equivalent (FTE) personnel in the pre-Lean period and compare it to the current size of the workforce involved in QI work. Due to insufficient details and lack of access to the complete set of personnel databases in each health region, we use an approximation as described in the next section.

## Results

### Economic cost of selected Lean activities

Using the methodology described in the previous section and the details about event participants’ time (Table 1) at each Lean event, we estimated the cost for a specific Lean activity and present them in Table 2.

Table 2 shows the cost for a typical Lean activity computed using average wage rates for Lean participants. In the Additional file 1: Tables S1-S7, we present our estimations for the cost of each event using three scenarios (low, average, and high) as described in Method section.

As shown in Table 2, there are variations in cost per event across different types of Lean tools. The cost ranges from $9337 to $76,927. The least expensive tools are Kaizen basics workshops and 5S, while the NAT is the most expensive event. However, the total numbers of each event need to be taken into account to evaluate their contributions to the total cost of Lean implementation. These total cost estimates are presented in Table 3.

**Table 3:** Total cost of Lean implementation in Saskatchewan (2012–2014)

		Cost in millions CAD$		
Lean activities	Numbers of events [1]	Low	Average	High
RPIWs	201	6.44	7.13	7.88
5S	80	0.81	0.89	0.96
Kaizen basic workshops	565	4.80	5.28	5.79
Mistake proofing projects	50	2.12	2.34	2.58
North American tour	17	1.20	1.31	1.43
Kanban	33	0.70	0.78	0.86
*Total for Lean events above*	*-*	*16.07*	*17.73*	*19.50*
Consulting fees (JBA contract)	-	19.56	19.56	19.56
Cost of new hires at KPOs/KOTs	-	8.64	9.60	10.56
Total for 2 years		44.27	46.89	49.62

Source: Provincial Kaizen Tracker database for the Lean events [23], and our computations for cost estimates

### Total cost of Lean for the Saskatchewan healthcare system

We report the total cost of Lean implementation in Saskatchewan in this section. The first column of Table 3 presents the total numbers of Lean events until April 2014. This information was compiled from the PKPO Kaizen database [23]. Although the database is updated on a regular basis, there is incomplete information for 5S events. At the time of our review of this database, there were thirty-three 5S events that took place from March to December 2013. Based on this information, we estimated that the total number of 5S events during the course of two years would be around 80. As indicated before, using the total number of individuals and average numbers of participants in Kaizen basics workshops and the NAT, we estimated the total number of corresponding events.

The table shows that the RPIWs and training workshops are the most frequently occurring activities implemented in Saskatchewan Lean transformation, followed by 5S events and mistake proofing projects. The table suggests that there were about eight RPIWs and 23 Kaizen basics workshops implemented across the province in a given month. However, one needs to interpret these numbers carefully given that there were limited numbers of events implemented in 2012 compared to more recently the. It is likely that the average number of events during 2014 would be higher.

Next, we estimated the total cost of each type of event during the course of Lean implementation until April 2014. For this, we used three scenarios. Under the low cost scenario, we assumed that the opportunity cost of Lean event participants’ time was equivalent to the lowest 25th percentile of the wage distribution for the corresponding job. Alternatively, we assumed that it was equivalent to the 75th percentile of the wage distribution under the alternative, high cost scenario. The third option (average) evaluated the opportunity cost of the participants’ time at the mean wage level for the corresponding job [25]. The total cost of each Lean event for each scenario was estimated and is presented in Table 3 using the corresponding unit cost for each event presented in Additional file 1: Tables S1-S7.

Table 3 shows the corresponding total costs for various Lean events, which suggests that the highest amount of resources are devoted to the RPIWs. The Kaizen basic workshops’ total cost of more than $5 million was higher than the total cost for all 5S events, mistake proofing projects and Kanban events, combined. Our estimates show that the total cost of implementing these Lean events ranges from $16 to $19.5 million depending on the assumption made for the value of participants’ time in these events. This implies that the annual cost of implementing these events at the same rate would be around $9 to $10 million. However, the actual cost depends on the number of events implemented and other types of Lean events that are taken into consideration. One could use unit cost of each event (Table 2) to compute the projected cost under various assumptions regarding the numbers of events that would be implemented.

In addition to the cost components mentioned above, there were also two additional sources of costs attributable to the Lean. The first was the consulting fees paid to the JBA as part of the contractual agreement with the JBA and the Ministry. According to the contract agreement, the consulting company agreed to deliver services to assist and to play a direct role in the implementation of the Lean, as well as to provide certification and training for the Lean specialists across the province. The details are documented in the contract agreement between the provincial government and the consulting company [34]. The original agreement was a 4-year contract with a total payment of $40 million. However, the government recently renegotiated the contract to end it after three years and four months with a total payment of $32.6 million. This total payment reported by the Government of Saskatchewan implies an average monthly payment of $815,000 over the course of the program, which is lower than the average monthly payment for the first two years of the contract. To compute the total consulting fees paid to the JBA, we used $815,000 as an average monthly payment for two years, which leads to a total payment of $19.56 million for a 2-year term (Table 3).

The second cost component was the expansion of QI teams in each health region due to the Lean initiative. This is, however, not straightforward to compute, given that the previous QIDs (or individuals engaged in QI work) in health regions were transformed to become the KPOs with QI personnel transferred to the new offices after the beginning of Lean. Although our primary interest is related to the additional costs, as compared to the pre-Lean period, among QI teams across the province, this information was not readily available to us. Therefore, we used the following approximation in our estimation of additional costs regarding the expansion of the QI labor force in the province.

We reviewed the human resources at the KOT/KPO at the SHR. Our choice of reviewing the SHR human resource database was determined by online availability of this information, the larger population of the health region, and the high numbers of Lean events completed in this health region compared to the rest of the province. Our review showed that the SHR has a Kaizen Operating Team (KOT), in addition to a KPO with a total of 25 Lean specialists, 4 KPO lead specialists, 8 leads (managers), 11 additional staff, and a director of the KPO [35]. The KOT is a new initiative solely associated with the Lean initiative; therefore, we included all KOT personnel costs as part of the additional costs of Lean. The question that remains is the proportion of KPO personnel solely associated with the Lean initiative. Based on our review of the names listed on the SHR Quarterly Review [35], we determined that about half of the personnel of the KPO were new employees, suggesting a total of 13 new hires (6 KPO specialists, 2 KPO lead specialists, 2 managers, 2 analysts, and 1 project coordinator). We used the same methodology to estimate the total wage and benefits paid for these personnel using average wages and benefits [25, 36]. Our estimations suggest that the annual personnel cost attributable to the Lean initiative is $2.40 million for the SHR (see Additional file 1: Table S7). We have no information for the rest of Saskatchewan; therefore, we assume that the associated cost in all other health regions, the PKPO, and the Ministry of Health is equivalent to the corresponding cost at the SHR. This suggests that the annual cost attributable to the expansion of QI teams in the rest of the province is $2.40 million. This results in a total 2-year cost of $9.6 million. We inflate (deflate) this total by 10% in order to calculate it for high (low) scenarios. We believe that this is a conservative assumption given that the rest of the province has a substantially larger population than the SHR.

Our estimates presented in Table 3 could underestimate the true cost of the Lean initiative in Saskatchewan. Due to lack of data, there are other components of associated costs that are not incorporated into our estimates. For instance, there are also costs associated with recruitment and retention of personnel. Given that Lean training is a complex and human resource intensive investment, retention of the trained personnel within the provincial healthcare system becomes especially valuable. If the healthcare system were to lose trained personnel, it would be an additional cost to the system since the new hires may need to be trained in Lean. The cost related to recruitment and retention can be estimated using the training cost and number of trained personnel who left the system. This issue is not taken into account in our estimates since we do not have information on the number of trained personnel who left the healthcare system during this period.

When we estimated the additional cost due to new hires to the KPOs/KOTs, we included only their salaries and benefits. It is plausible to expect that there were additional operational costs associated with new hires. Office space, utilities, and supplies would be some examples of additional costs due to expansion of QI teams in the province that were not included in our cost estimations.

Given that the Lean implementation required substantial resources to be directed for its activities, the regions with fewer resources and less capacity have had considerable difficulty in delivering their day-to-day clinical or non-clinical goals [30]. Proportionately, smaller and less well-resourced health regions reported in the qualitative interviews [30] feeling disadvantaged and having experienced a greater burden from the implementation of Lean compared to their counterparts with larger budgets. It is likely that resource allocation for the Lean implementation will lead to fewer services being delivered in these regions. We cannot estimate these without collecting relevant data.

## Discussion

Our cost estimates show that the annual cost of Lean in Saskatchewan during the 2012–2014 period was about $23.4 million, based on our estimates using data from 2012 to 2014 implementation period. Without the consulting fees, its annual cost would be around $13.6 million (see Table 3). The annual cost of Lean implementation during subsequent years will differ from these estimates given that the intensity and type of Lean activities would not be the same. Using the average cost estimates for selected Lean events (see Table 2), one could compute Lean implementation costs for the subsequent years.

These estimates presented above are conservative, given the limited data. For instance, it is known that the senior leaders across the province have participated in Lean Leadership training, but we do not have exact information on their participation in the corresponding activities. These individuals are listed with other managers as Lean leader participants in project forms for the corresponding activity; therefore, we used government managers’ wage rates for their time, recognizing that these are substantially lower wage rates than those for the senior leaders. There are other Lean activities not included in our estimates. Some examples would be training components of Lean leader training that were not captured by the selected events in our computations (e.g., value stream mapping followed by the training modules – modules deep-dive and module marathons). Other examples would be Hoshin Kanri meetings that take place three times a year and require attendance from senior leaders and sometimes the directors of health regions.

There are ways that this research may inform the policy at this stage. These findings are supported by our cost estimations, which show that there are substantial variations in total cost for each type of Lean activity. It is possible that there are alternative ways to carry out these events with some efficiency gains. Following the first implementation phase of Lean, there have been some changes to the training sessions. As of July 2015, the Kaizen basics workshop was redesigned so that the event takes one half-day rather than one full-day. This modification would decrease the cost by about $2.5 million in two years. One needs to consider the changes while computing the ongoing costs of the Lean implementation in Saskatchewan.

There are also possible variations in returns from different Lean tools. For instance, mistake proofing projects may have immediate health impacts compared to training workshops. At the same time, there might be settings or practice areas where the return is higher for the same Lean tools. Based on our preliminary review of mistake proofing projects, we identified three mistake proofing projects on mislabelling of blood tests or specimens and another three projects on fall prevention. The results from these projects, as reported in the project forms, showed that the mistake proofing projects have been extremely successful in fall prevention with a 100% reduction in falls, but the same degree of success has not been observed in mislabelling projects. Out of three mislabelling projects that we reviewed, the errors decreased by about 80–90% in two projects, but errors increased by about 66% in the other. This preliminary review shows that there might be substantial gains from increasing the intensity of the same Lean activity in certain areas in which the return is higher. These areas with high return need to be identified on a regular basis with ongoing and timely analysis by using the Lean database, which should include reliable and consistent measures across various aspects of the healthcare system.

## Conclusions

The cost reported in this paper is due to new resources devoted to the QI efforts through the Lean initiative compared to the pre-Lean period. In order to assess the return from this investment, the cost needs to be compared to the additional benefits incurred in the form of efficiency gain and waste reduction, as well as improvement in health outcomes. It is likely that some benefits would be observed in the short term (e.g., waste reduction, efficiency gain), while others, such as a decrease in mortality rates, may require a much longer term to be achieved. With this paper, we only offer estimates for the cost of this investment without comparing it to the benefits. A complete evaluation should be carried out based on a comprehensive economic evaluation approach in order to examine the return from this Lean investment for Saskatchewan. At this point, we do not have enough information to make such an evaluation.

Some activities mentioned in this paper were redesigned or discontinued after our study period. As previously mentioned, as of July 2015, the re-crafted Kaizen basics workshops changed from a full day to one half day event. The NAT, which was a required part of mistake proofing, has been put on hold. This activity is being reassessed as it has not been seen as a requirement to achieve Lean leader certification. Time commitment by the staff for RPIWs has also decreased because this process has been streamlined and requires Lean leaders to participate once rather than twice in an event. One needs to take these changes into account in order to estimate the cost of Lean after the first 2-year period evaluated in this paper.

## Additional file

Additional file 1: Detailed computation of cost for each Lean event. **Table S1**. Cost for an RPIW. **Table S2.** Cost for a 5S activity. **Table S3.** Cost for a Kaizen Basic Workshop. **Table S4.** Cost for a Mistake Proofing Project. **Table S5.** Cost for a North American Tour. **Table S6.** Cost for a Kanban event. **Table S7.** Annual cost of Lean at Saskatoon Health Region due to KPOs/KOTs. (PDF 215 kb)

## Abbreviations

5S: Sorting, simplifying, sweeping, standardizing and self-discipline
CAD: Canadian dollars
CPP: Canadian pension plan
EI: Employment insurance
FHHR: Five hills health region
FTE: Full time equivalent
JBA: John Black and associates
JIT: Just-in-time
KOT: Kaizen operating teams
KPO: Kaizen promotion office
NAT: North American Tour
PAPHR: Prince Albert Parkland Health Region
PKPO: Provincial Kaizen promotion office
QI: Quality improvement
QIDs: Quality improvement departments
RPIW: Rapid process improvement workshops
RQHR: Regina Qu’Appelle Health Region
RRSP: Registered retirement saving plan
RTC: Releasing time to care
SHR: Saskatoon health region

## Footnotes

1 Since the records related to job titles and affiliations of the participants in Kaizen basic workshops are incomplete, for costing we used the average wage rate in the healthcare sector for the participants in the workshops.

2 The hourly wage rate for government managers including benefits and other contributions paid by the employers is $51.69 (25th percentile), $56.98 (average), and $63.05 (75th percentile) [25].

3 In the case of CPP, there is an exemption of $3500.

4 The minimum annual workers’ compensation premium is $100 and the maximum assessable wage is $55,000 [29].

5 These are the most common employer contributions paid by public employers in Saskatchewan.

6 There is no information for the printing and supplies for any events. Given the number of the participants in these events, we believe that our assumption for the cost of printing and supplies is reasonable. As shown in Table 2, the cost of printing and supplies ($100 or $200) is negligible as compared to the total cost of the corresponding event ($9337 to $35491). Therefore, our total cost estimates are not sensitive to this assumption.

7 In the Additional file 1: Tables S1-S7, we present three different levels of estimates by inflating (deflating) this airfare by 10% for a high (low) scenario.
