# Supplementary material for: An economic analysis of a system wide Lean approach: cost estimations for the implementation of Lean in the Saskatchewan healthcare system for 2012–2014
Source: BMC Health Serv Res. 2017 Aug 3;17:523. doi: 10.1186/s12913-017-2477-8 (PMC5543735; doi:10.1186/s12913-017-2477-8)
Supplement: Additional file 1: — Detailed computation of cost for each Lean event. Table S1. Cost for an RPIW. Table S2. Cost for a 5S activity. Table S3. Cost for a Kaizen Basic Workshop. Table S4. Cost for a Mistake Proofing Project. Table S5. Cost for a North American Tour. Table S6. Cost for a Kanban event. Table S7. Annual cost of Lean at Saskatoon Health Region due to KPOs/KOTs. (PDF 215 kb) [file 12913_2017_2477_MOESM1_ESM.pdf]

Supplementary material

An economic analysis of a system wide Lean approach: Cost estimations for the implementation of Lean in the Saskatchewan healthcare system for 2012-2014

Nazmi Sari, PhD[1]
Department of Economics, University of Saskatchewan

Thomas Rotter, PhD
College of Pharmacy & Nutrition, University of Saskatchewan

Donna Goodridge, PhD
College of Medicine, University of Saskatchewan

Liz Harrison, PhD
College of Medicine, School of Physical Therapy, University of Saskatchewan

Leigh Kinsman, PhD
University of Tasmania and Tasmanian Health Organisation (North

[1] *Corresponding author:* Nazmi Sari, Department of Economics, University of Saskatchewan, Arts 815, 9 Campus Drive, Saskatoon, SK, Canada S7N 5A5; E-mail: Nazmi.Sari@usask.ca; homepage.usask.ca/~sari/

Table S.1: Cost for an RPIW

		**Cost in current Canadian dollars**		
	Total number of hours	Low	Average	High
Team Lead	112.5	5699	6282	6952
Sub-team Lead	103.12	5224	5759	6372
KPO support	159.37	7991	8809	9748
Lean Leader Participant	45.69	2362	2604	2881
Physician	15.51	2909	2909	2909
Patient/Family Representative	40.08	374	721	1069
Other Team Members	156.46	5428	5907	6342
Printing/supplies [1]	n/a	150	200	250
Travel/accommodation [2]	n/a	1125	1500	1875
Overtime compensations	n/a	800	800	800
TOTAL		$32,062	$35,491	$39,198

Note: It is assumed that there is one team lead, sub-team lead and a KPO support person in each event. [1] We assumed that the cost of printing and supplies is incurred in week -3 and the event week with an average weekly cost of $100. Then we inflated (deflated) it by 25% for high (low) scenario. [2] There might be travel, accommodation, parking, and other incidental expenses. We assumed an average of one out-of-region person who will be travelling for the event with an average cost of $1,500, that was inflated (deflated) by 25% for high (low) scenario. n/a stands for not applicable.

Table S.2: Cost for a 5S activity

		Cost in current Canadian dollars		
	Total number of hours	Low	Average	High
Team Lead	37.5	1907	2137	2365
Sub-team Lead	37.5	1907	2137	2365
KPO support	37.5	1907	2137	2365
Lean Leader Participant	1.04	54	60	66
Physician	3.13	587	587	587
Other Team Members	98.95	3442	3735	3981
Printing/supplies	n/a	75	100	125
Overtime compensations	n/a	272	272	272
TOTAL		$10,076	$11,165	$12,001

Note: It is assumed that there is one team lead, sub-team lead and a KPO support person in each event. We assumed that the cost of printing and supplies is $100. Then we inflated (deflated) it by 25% for high (low) scenario. n/a stands for not applicable.

Table S.3: Cost for a Kaizen Basic Workshop

		**Cost in current Canadian dollars**		
	Number of people	Low	Average	High
Workshop Participants	30	7196	7923	8730
Physician	0.43	484	484	484
Facilitator/instructor	1	335	381	427
Travel/accommodation	n/a	113	150	187
Printing/supplies	n/a	75	100	125
Overtime compensations	n/a	299	299	299
TOTAL		$8,502	$9,337	$10,252

Note: Based on our review of workshops across health regions and other healthcare providers, we determined that the average number of participants in each workshop is about 30 people per session. The workshop takes one day (7.5 hours). Average wages for workshop participants are assumed to be the provincial average in healthcare sector. The KPO person is assumed to be a Kaizen Lead Specialist with monthly salary range of $5,752- $7,476 (Government of Saskatchewan, 2014a). There are 16,963 individuals (including 244 physicians) participated in the workshops in the period of April 2012 and March 2014. The physician remuneration is $187.50 per day (Government of Saskatchewan, 2014b). n/a stands for not applicable.

Table S.4: Cost for a Mistake Proofing Project

		Cost in current Canadian dollars		
	Number of hours	Low	Average	High
Team Lead	120	6203	6838	7566
Sub-team Lead	120	6203	6838	7566
Team members	562	29029	32000	35409
Overtime compensations	n/a	1016	1016	1016
TOTAL		$42,451	$46,692	$51,557

Note: Average number of team members for a typical event is 4.8 individuals. Each team member is expected to participate 117 hours during the course of the event. The wage rate for each participant is assumed to be the same with the government managers' wage in healthcare sector. These are \$51.69 (25th percentile), \$56.98 (average), and \$63.05 (75th percentile). n/a stands for not applicable.

Table S.5: Cost for a North American Tour

		Cost in current Canadian dollars		
	Descriptions	Low	Average	High
Participants	20 individuals	38768	42735	47286
Travel	Air (Saskatoon-Salt Lake City-Seattle)	11280	12420	13760
Accommodation	5 nights/person	13000	14000	15000
Per-diem	6 days/person	6120	6120	6120
Local transportation	Travel to the airports	1500	1500	1500
Overtime compensations	See text	152	152	152
TOTAL		$70,820	$76,927	$83,818

Note: Based on our communication with the PKPO, we assumed total number of participants to be 20 for each NAT. As a part of the tour, they spent time in Salt Lake City for one day followed by 4 days stay in Seattle (United States). The wage rate of the participants is assumed to be of Government managers in health care (Government of Saskatchewan, 2013). Airfare is computed at the economy rate with the low airfare of $564 per person, average of $621 per person, and high fare of $688 per person. The accommodation per night is calculated as low as $130 per person, on average $140/ person and high as $150/person. The per diem rate is obtained from Canada Revenue Agency (2014).

Table S.6: Cost for a Kanban event

		Cost in current Canadian dollars		
	Number of hours	Low	Average	High
Team Lead	90	4652	5128	5675
Sub-team Lead	90	4652	5128	5675
KPO Support	75	2876	3308	3738
Coordinator	53	1241	1477	1593
Team members	226	7216	7958	8667
Overtime compensations	n/a	676	676	676
TOTAL		$21,313	$23,675	$26,024

Note: Average number of team members for a typical event is 6.03 individuals. Each team member is expected to participate 37.5 hours during the course of the event. We used the manager wage rates for team lead and sub-team leads, and average wage in health care sector for the team members. For the coordinator and the KPO support personnel, we used the corresponding wage rates for these job titles. The wage rate for each participant is assumed to be the same with the government managers' wage in healthcare sector. These are \$51.69 (25th percentile), \$56.98 (average), and \$63.05 (75th percentile) (Government of Saskatchewan, 2013). The cost related to the sensei, and the interpreter for the sensei has not been included in our estimates above. n/a stands for not applicable.

Table S.7. Annual cost of Lean at Saskatoon Health Region due to KPOs/KOTs

	Number of new hires	personnel cost
Manager	6	666,616
KPO Lead specialist	2	194,376
KPO/KOT specialist	18	1,345,734
Analyst	2	137,319
Coordinator	1	54,346
Total	29	2,398,391

Notes: We used average wage and benefit rates for all personnel in this table. KPO Lead specialists are assumed to be paid at the upper bound of KPO Lead specialist pay range while the KPO/KOT specialists are assumed to be paid at the lower bound. Analysts are assumed to be paid at the average rate for healthcare sector.

References:

Canada Revenue Agency. 2014. Meal and Vehicle rates used to calculate travel expenses for 2013 and previous years. http://www.cra-arc.gc.ca/tx/ndvdls/tpcs/ncm-tx/rtrn/cmpltng/ddctns/lns248-260/255/rts-eng.html. Accessed 2 July 2014.

Government of Saskatchewan. 2014. Kaizen Lead Specialist- CON000888. (job posting Feb, 2014)

Government of Saskatchewan. 2013. Saskatchewan Wage Survey Report 2011. http://ae.gov.sk.ca /sk wage-survey-report-2011. Accessed 6 March 2013.
